# Neural correlates of spatial and nonspatial attention determined using intracranial electroencephalographic signals in humans

**DOI:** 10.1002/hbm.23225

**Published:** 2016-04-29

**Authors:** Ga Young Park, Taekyung Kim, Jinsick Park, Eun Mi Lee, Han Uk Ryu, Sun I. Kim, In Young Kim, Joong Koo Kang, Dong Pyo Jang, Masud Husain

**Affiliations:** ^1^ Department of Neurology University of Ulsan College of Medicine Seoul Korea; ^2^ Department of Biomedical Engineering Hanyang University Seoul Korea; ^3^ Department of Neurology University of Ulsan College of Medicine, Ulsan University Hospital Ulsan Korea; ^4^ Nuffield Department of Clinical Neurosciences University of Oxford Oxford United Kingdom

**Keywords:** intracranial electroencephalography, nonspatial attention, parietal lobe, spatial attention, time–frequency analysis

## Abstract

Few studies have directly compared the neural correlates of spatial attention (i.e., attention to a particular location) and nonspatial attention (i.e., attention to a feature in the visual scene) using well‐controlled tasks. Here, we investigated the neural correlates of spatial and nonspatial attention in humans using intracranial electroencephalography. The topography and number of electrodes showing significant event‐related desynchronization (ERD) or event‐related synchronization (ERS) in different frequency bands were studied in 13 epileptic patients. Performance was not significantly different between the two conditions. In both conditions, ERD in the low‐frequency bands and ERS in the high‐frequency bands were present bilaterally in the parietal cortex (prominently on the right hemisphere) and frontal regions. In addition to these common changes, spatial attention involved right‐lateralized activity that was maximal in the right superior parietal lobule (SPL), whereas nonspatial attention involved wider brain networks including the bilateral parietal, frontal, and temporal regions, but still had maximal activity in the right parietal lobe. Within the parietal lobe, spatial attention involved ERD or ERS in the right SPL, whereas nonspatial attention involved ERD or ERS in the right inferior parietal lobule. These findings reveal that common as well as different brain networks are engaged in spatial and nonspatial attention. *Hum Brain Mapp 37:3041–3054, 2016*. © **2016 The Authors Human Brain Mapping Published by Wiley Periodicals, Inc.**

## INTRODUCTION

Attention is the cognitive process that selectively focuses on or ignores separable information to allow us to accomplish our immediate goals [Gunduz et al., 2011]. During the attention process, we can selectively allocate limited mental resources to a variety of pieces of information, such as a location or a particular feature in the visual scene [Maunsell and Treue, 2006]. Both spatial and nonspatial featured‐based attention can be used to optimize allocation of selective attention [Egner et al., 2008].

Several brain areas are involved in spatial and nonspatial attention. The posterior parietal cortex is considered to have an important role in spatial cognitive processes [Culham and Kanwisher, 2001; Nachev and Husain, 2006]. Patients with hemispatial neglect due to damage to the right parietal lobe exhibit a decrement in vigilance over time, often used as an indicator of a sustained attention deficit, while performing a spatial attention task, but not while performing a nonspatial attention task [Malhotra et al., 2009], suggesting that the cortical substrates for spatial and nonspatial attention might be different. However, several studies have argued that spatial and nonspatial attention rely on closely related mechanisms [Maunsell and Treue, 2006], reporting that the foci of frontoparietal activation in nonspatial cueing tasks were very similar to those during spatial attention tasks [Corbetta et al., 2000; Hopfinger et al., 2000].

Some investigators have also proposed that different parts of the human parietal lobe have different roles in spatial and nonspatial attention [Husain and Nachev, 2007; Nachev and Husain, 2006; Schenkluhn et al., 2008], with the inferior parietal lobule (IPL) playing a role in tasks that are nonspatial or tasks that are not necessarily spatially lateralized [Husain and Rorden, 2003; Nachev and Husain, 2006]. By contrast, the superior parietal lobe (SPL) or intraparietal sulcus may play a role in the allocation of spatial attention and visually guided movement to spatial locations [Culham and Valyear, 2006; Nachev and Husain, 2006]. Thus, the anatomical substrates involved in spatial and nonspatial attention need to be elucidated. In particular, it remains to be determined whether the two attention systems use the same brain substrates or how much activation there is in different brain areas during spatial and nonspatial attention tasks.

Many functional magnetic resonance imaging (MRI) studies have shown regional characteristics dependent on the attention task [Greenberg et al., 2010; Tana et al., 2010]. However, functional imaging studies are not able to demonstrate instantaneous electrophysiological changes, which may reveal different oscillation characteristics in the time and frequency domains [Pfurtscheller and Lopes da Silva, 1999]. The intracranial electroencephalography (iEEG) signal offers a high temporal resolution and high signal fidelity and is useful for revealing oscillatory brain activity during attention tasks [Gunduz et al., 2011]. Changes in the oscillations of different brain areas and in the distribution of cortical activity during spatial and nonspatial attention tasks remain to be elucidated. Evaluation of time–frequency patterns and identification of differences in cortical distribution maps between spatial and nonspatial attention tasks may help us to understand the dynamic changes that occur during these tasks but cannot be revealed by functional imaging studies.

To summarize, the aim of this study was to investigate the neural correlates of two different types of attention processing. We evaluated the power change in the iEEG signal during spatial and nonspatial attention tasks and compared spatiotemporal brain activity over several spectral bands between the two tasks in 13 epileptic patients who had implantations of intracranial grid electrodes. This study provides important information on the dynamic nature of both attention mechanisms.

## MATERIALS AND METHODS

### Characteristics of Human Patients

Thirteen epileptic patients (six females; mean age 31.3 ± 11.8 years) who were undergoing invasive studies for epileptic surgery that used intracranial electrodes such as subdural grids and strips participated in this experiment. This study was approved by the Asan Medical Center, Seoul, Korea. Informed consent was obtained in accordance with the regulations of the Research Ethics Board of our institution. All patients had language dominance in the left hemisphere confirmed by an intra‐arterial amobarbital test. Of the 13 patients, six had left hemispheric onset epilepsy and seven had right hemispheric onset epilepsy. The clinical information of the participants is summarized in Table 1.

**Table 1:** Participant characteristics

ID	Sex/age	Electrode (number of electrodes)	Diagnosis	Handedness	Language dominancy	MRI
A	M/31	Rt. Ant. F G (64), Rt. Post. F G (32)	Rt. FLE	Right	Left	Normal
B	M/21	Rt. P G (16), Rt. Sup. F G (8), Rt. Mid. F G (8)	Rt. FLE	Right	Left	Normal
C	F/29	Rt. F S (4), Rt. F G (20), Rt. P S (4), Rt. P G (32)	Rt. F‐PLE	Right	Left	Tumors in right frontal and parietal lobes
D	M/32	Rt. F G (32), Rt. O F G (16)	Rt. TLE	Right	Left	Normal
E	F/26	Rt. F G (28), Rt. Inf. F G (16)	Rt. FLE	Right	Left	Nonspecific focal HSI in the right dorsolateral parietal subcortex
F	M/39	Rt. P G (32)	Rt. PLE	Right	Left	Normal
G	F/15	Rt. Sup. F G (32), Rt. Inf. F S (8), Rt. Mid. F S (4), Rt. F S (4)	Rt. FLE	Left	Left	Cortical dysplasia in the right frontal area
H	F/41	Lt. P G (16)	Lt. TLE	Right	Left	Left hippocampal atrophy
I	F/26	Lt. Inf. P G (31), Lt. Post. P G (20), Lt. Ant. P G (8)	Lt. TLE	Right	Left	Normal
J	M/16	Lt. T G (16)	Lt. TLE	Left	Left	Normal
K	F/30	Lt. F P G (32), Lt. Ant. F G (20)	Lt. FLE	Right	Left	Left frontal tumor
L	M/44	Lt. F G (12), Lt. Inf. F G (32), Lt. Ant. T G (20), Lt. Post. T G (16)	Lt. TLE	Right	Left	Post‐traumatic encephalomalacia, left frontotemporal
M	M/26	Lt. F G (48), Lt. O F G (32), Lt. F P G (16)	Lt. FLE	Right	Left	Normal

MRI: magnetic resonance imaging; Lt.: left; Rt.: right; Ant.: anterior; Post.: posterior; Sup.: superior; Inf.: inferior; Mid.: middle; F: frontal; P: parietal; T: temporal; O: orbital; G: grid; S: strip; FLE: frontal lobe epilepsy; F‐PLE: frontoparietal lobe epilepsy; PLE: parietal lobe epilepsy; TLE: temporal lobe epilepsy.

All patients had a noninvasive presurgical work‐up that included volumetric brain MRI, fluorodeoxyglucose positron emission tomography (FDG‐PET), and scalp electroencephalography (EEG) monitoring. Because of discrepancies in the results of these noninvasive studies, all patients presented here also underwent intracranial electrode insertion under a clinical protocol to better define the epileptic foci. The location of the subdural electrode implantation (Ad‐Tech Medical Instrument Corp., Racine, WI) was determined according to the results of each patient's noninvasive work‐up. The implanted electrode grids consisted of electrodes that were 4 mm in diameter, spaced at an interelectrode distance of 1 cm, and embedded in silicone.

After finishing a video‐monitoring study to localize the seizure foci with intracranial electrodes, seizures were controlled by anticonvulsant medications. The experimental paradigm for this study was conducted approximately 5–7 days after the electrode implantation, by which time all patients were well enough to perform these experiments.

### Experimental Paradigm

The spatial and nonspatial attention tasks were developed using VIZARD software (World Viz Inc.) and adopted from a paradigm that examined the spatial (location‐based) and nonspatial (feature‐based) attributes of the same pattern stimuli [Malhotra et al., 2009]. The key difference between the spatial and nonspatial attention tasks was that the nonspatial task required attention to be directed to the identity of the patterns rather than their locations, whereas the spatial task required attention to be directed to the locations of the patterns, regardless of their identity. Thus, any difference in performance between the tasks would not be due to the requirement of attending to different attributes of the tasks [Malhotra et al., 2009].

The tasks were performed in a quiet room that contained the EEG equipment. Participants were presented with the attention task paradigm while sitting in a comfortable chair 50 cm in front of a laptop screen. They were asked to respond as quickly as possible by pressing the space bar on a keyboard with their right hand when they saw predefined target stimuli (target response). They were asked not to press the space bar on the keyboard when they saw nontarget stimuli (nontargets).

**Figure 1:**
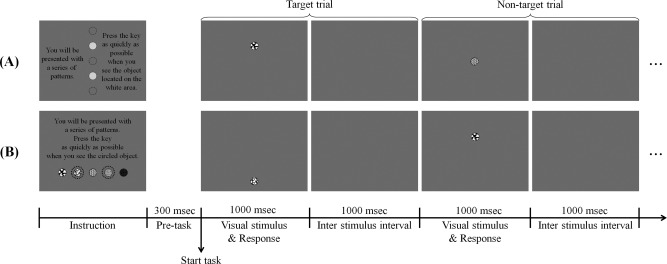
Spatial and nonspatial attention task design. **(A)** In the spatial attention task, participants were instructed to respond when a stimulus was presented at one of the two predefined locations (indicated here by arrows for display purposes). **(B)** In the nonspatial attention task, participants were instructed to respond when the pattern of the stimulus matched one of the two predefined target patterns (shown circled here), regardless of its spatial location. In both tasks, the first test display shows the target location or pattern and the third test display shows the nontarget location or pattern.

During both spatial and nonspatial attention tasks, circular visual stimuli were presented on a uniform gray background. In each trial, one of the five different patterns was presented on one of the five different positions along the vertical meridian of the screen as a stimulus (Fig. 1). The stimulus was presented every 2 s and remained on the screen for 1 s. Over a total period of 15 min, 500 stimuli were presented: 200 target stimuli and 300 nontarget stimuli. Over the 500 trials, every pattern and position was presented an equal number of times. The same set of stimuli was used for the spatial and the nonspatial attention tasks. The order of the two tasks was randomly selected for each participant to control order effect.

For the spatial attention task, the participants were asked to respond as quickly as possible when a stimulus appeared at one of two predesignated target locations, indicated by solid circle in Figure 1A. In the nonspatial attention task, participants were instructed to respond as quickly as possible when the pattern of the stimulus matched one of two predefined target patterns, regardless of its spatial location (Fig. 1B). The two tasks were performed in a random order, separated by a 5 min rest. After a brief practice session, participants were asked to press the space bar key on the keyboard and begin the testing session when they felt that they fully understood the tasks.

### iEEG Data Recording

During the experiment, iEEG signals were recorded continuously at a sampling frequency of 1000 Hz and referenced to the Pz electrode on the scalp with a Stellate Harmonie System (Stellate, Montreal, Canada). No hardware filters were used. All signals were recorded throughout each task.

### Behavioral Data Analysis

The response time and correct hit rate were measured for both tasks. Data were analyzed with SPSS 21.0 (SPSS Inc., Chicago, IL) and compared across tasks (spatial attention, nonspatial attention) using a Wilcoxon rank‐sum test at the two‐tailed *P* value of 0.05 (uncorrected).

### iEEG Data Processing

MATLAB (Version 2010b; MathWorks, Natick, MA) and EEGLAB toolbox (Version 9; Swartz Center for Computational Neuroscience, La Jolla, CA) were used to process iEEG data. iEEG signals were processed with a band‐pass filter from 1 to 200 Hz and were rereferenced to a common average reference [Gunduz et al., 2011]. An independent component analysis was performed to remove artifact components such as eye and muscle movements. Only electrodes that showed no or rare interictal epileptiform discharges were included in the analysis. Data were segmented into 2000 ms epochs from 500 ms before the appearance of the stimulus to 1500 ms after the appearance of the stimulus. Noisy epochs were then manually removed.

In each 2000 ms epoch, spectral analysis was performed using the short‐time Fourier transform, which is one of the most widely used signal‐analysis methods. Briefly, the short‐time Fourier transform divides the iEEG signal into small sequential or overlapping time segments, analyzes each time segment, and provides a time–frequency distribution [Kiymik et al., 2005]. We used a 500 ms sliding Hann window to obtain 200 time bins with a 7.5 ms shift between the bins. The analysis focused on 200 time bins × 149 frequency bins (2–150 Hz) × number of electrodes × number of trials.

Time–frequency analysis with event‐related spectral perturbation (ERSP) provides event‐related changes in the power of each frequency band. Such changes in spectral power reflect non‐phase‐locked changes in the activity of underlying neuronal populations, which can be cancelled out in trial‐averaged measures such as event‐related potentials [Tsuchiya et al., 2008]. A transient change in the power of a given frequency band is called event‐related synchronization (ERS) or event‐related desynchronization (ERD) according to whether it reflects an increase or decrease, respectively, in the synchrony of the underlying neuronal populations. ERD/ERS may occur due to changes in parameters that control oscillations in neuronal networks and can be viewed as being generated by changes in the activity of local interactions that control the frequency of the ongoing EEG [Pfurtscheller and Lopes da Silva, 1999].

To evaluate statistical changes in ERD and ERS over time during spatial and nonspatial attention tasks, we computed the ERSP for each trial for each electrode and frequency with a Bonferroni corrected *t*‐statistic of *P* < 0.05. Spectral iEEG features in every 200 ms window with an overlap of 100 ms from stimulus onset to 700 ms post stimulus onset were evaluated in theta, alpha, beta, low gamma, and high gamma bands (4–7, 8–13, 13–30, 30–50, and 70–150 Hz, respectively). The spatiotemporal course of the ERSP was expressed relative to baseline (the 500 ms before stimulus onset). ERD was quantified in low‐frequency bands (theta, alpha, and beta) and ERS was quantified in high‐frequency bands (low gamma and high gamma).

### Extraction of Electrode Locations and Transfer to Template Magnetic Resonance Images

**Figure 2:**
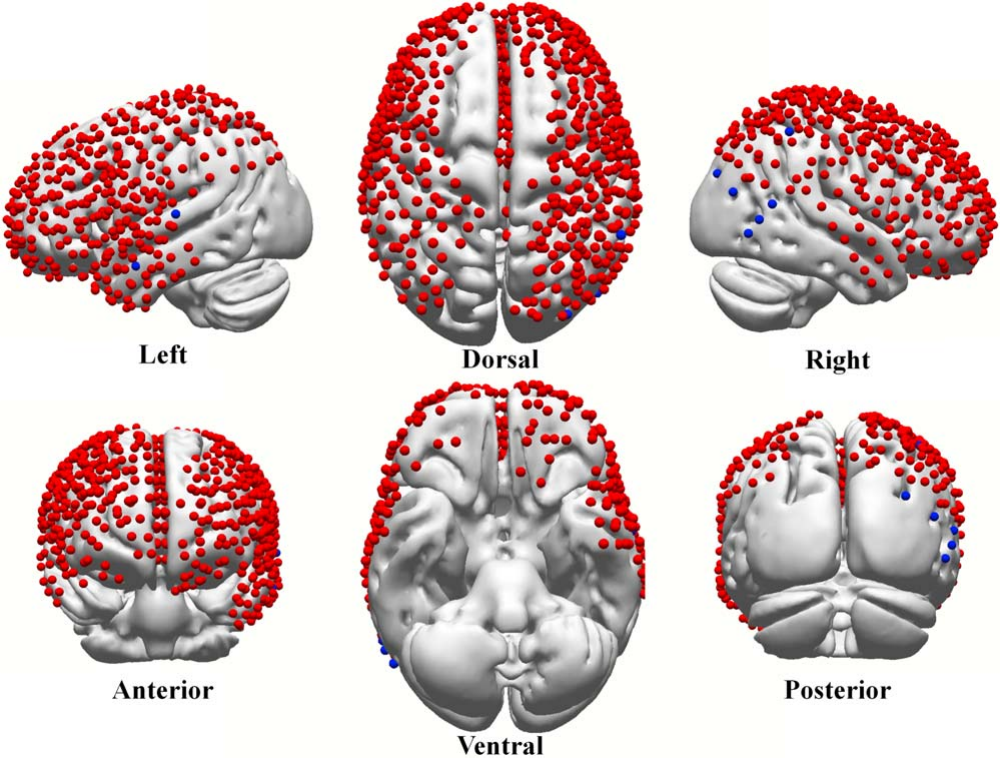
Locations of implanted grids on a standard cortical model resulting from coregistration of preoperative magnetic resonance images and postoperative computed tomography images. The locations of electrodes for all participants were identified by preoperative T1 magnetic resonance imaging data and postoperative three‐dimensional computed tomography data coregistration. The locations extracted for each participant were normalized and projected on the Montreal Neurological Institute standard model. The blue dots indicate the electrodes excluded because of frequent epileptic discharge. [Color figure can be viewed in the online issue, which is available at http://wileyonlinelibrary.com.]

The locations of the electrodes were identified on the basis of images registered between preoperative T1 MRI data and postoperative three‐dimensional computed tomography data using the FMRIB software library (http://www.frmib.ac.uk/fsl). The locations of each electrode were transformed into the Talairach coordinate system using Curry software (Compumedics, Charlotte, NC) and projected onto the template brain provided by the Montreal Neurological Institute (Fig. 2). The total number of electrodes was 528 and Table 2 displays the number of electrodes included in each area. The fractional change in oscillatory power in each of the five frequency bands was calculated for each electrode and the change in power was displayed using a color map. We did not include the delta band in this study due to the small time epoch.

**Table 2:** The number of electrodes and participants contributing to results in each region

	Left		Right	
	Number of electrodes	Number of participants	Number of electrodes	Number of participants
Frontal	166	7	186	7
Inferior parietal	20	3	30	3
Superior Parietal	7	1	41	4
Temporal	56	4	22	2

**Figure 3:**
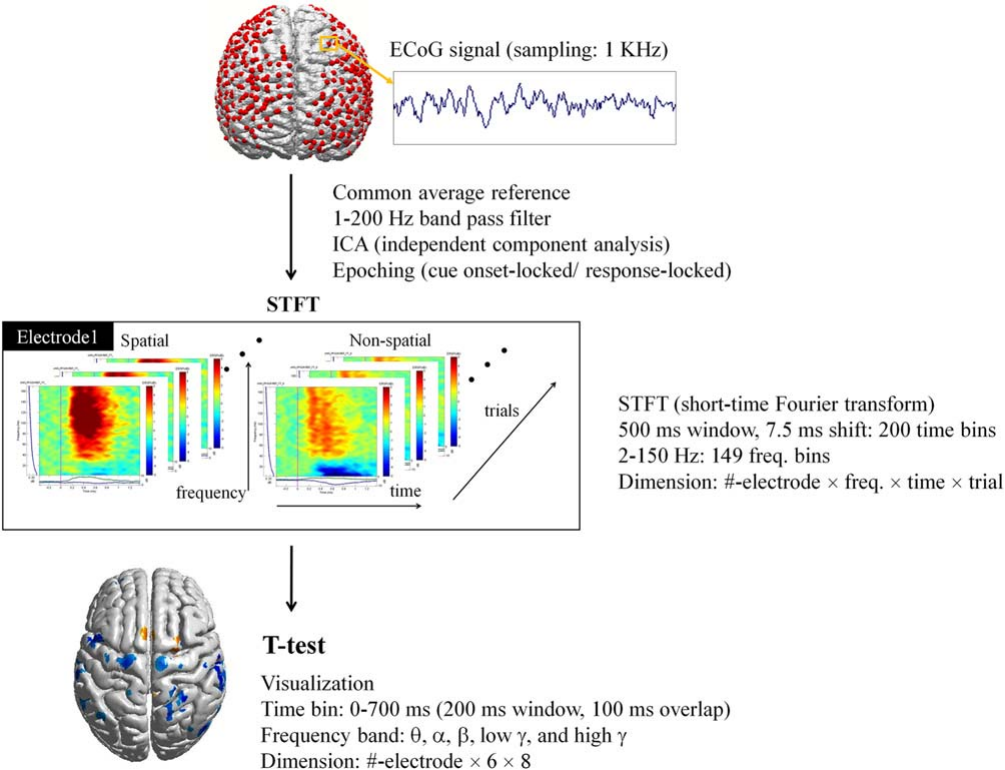
Schematic design of the analysis. The ECoG signal was sampled at 1 kHz and conditioned using filters and ICA. The filtered signal was divided into epochs for the time–frequency analysis using a 500 ms sliding window with a 7.5 ms shift between epochs. For statistical calculation and visualization, data were reduced into eight time bins (200 ms window, every 100 ms interval from 0 to 700 ms after stimulus onset) and five frequency bands (4–7, 8–13, 13–30, 30–50, and 70–150 Hz). [Color figure can be viewed in the online issue, which is available at http://wileyonlinelibrary.com.]

A nearest‐neighbor method was used to construct the color map. A cortical triangular mesh was colored relative to the statistical significance level of the closest electrode and the color faded as the distance between the mesh and the closest electrode increased [Englot et al., 2010; Youngblood et al., 2013]. The process of the analysis is illustrated in Figure 3.

### Electrode Counting

To show the task‐related differences across multiple participants in any region of interest, we adapted the individual electrode analysis method [Burke et al., 2013; Sederberg et al., 2007; van Vugt et al., 2010]. Briefly, this method shows the fraction of electrodes with a significant decrease (ERD) or increase (ERS) in oscillatory power in the same brain region for each task. Thus, we computed the change in oscillatory power at each time window for each electrode in eight regions of interest (frontal, superior parietal, inferior parietal, and temporal lobes for both hemispheres) defined using Talairach coordinates. Oscillatory power and its statistical changes with attention engagement in the five frequency bands were computed as described above. In addition, the number of electrodes showing significant ERD or ERS relative to baseline (at the statistical level of *P* < 0.05 corrected with the Bonferroni method for multiple comparisons) was counted for each region of interest, and expressed as a fraction of the total number of electrodes in that region of interest.

For each region of interest, the fraction of electrodes showing a significant ERD or ERS was compared between the two tasks. In addition, for each task, the fraction of electrodes showing a significant ERD or ERS was compared between the left and right hemispheres and between the right SPL and IPL. These tests were performed using the chi‐square test or Fisher's Exact test with a significance level of uncorrected *P* < 0.05.

### Temporal Topographic Maps

Initially, we aggregated the results of all electrodes in each region of interest for each participant. We then combined these results across participants using the template cortical model. Temporal topographic maps from 0 to 800 ms after stimulus onset were computed using the statistical results of the time–frequency analysis to visualize and compare the topographic differences between the spatial and nonspatial attention tasks. ERSP maps were created from −400 to +1400 ms relatively to the stimulus onset to reveal power changes during the task in each electrode. We focused on a time window of 400–600 ms after stimulus onset because cortical distribution mapping of spatiotemporal dynamics showed significant event‐related responses at the time window in both spatial and nonspatial attention tasks. We also compared the ERSP responses in the motor cortex to those outside the motor cortex, such as in the parietal, frontal, and temporal lobes, from stimulus onset or from response onset to investigate whether iEEG signals related to spatial or nonspatial attention were linked to the motor response.

### Differences in the Cortical Map between Spatial and Nonspatial Attention Tasks

Finally, we compared the fraction of electrodes that showed a significant increase or decrease in oscillatory power in the 400–600 ms period compared with the baseline interval at the statistical threshold of *P* < 0.05 (Bonferroni correction for multiple comparisons) during only one of the two tasks. We also compared cortical maps between the right SPL and IPL to compare the relation between the SPL and IPL for spatial and nonspatial attention.

## RESULTS

### Behavioral Performance

The average response time of all 13 participants was 0.86 ± 0.21 s for the spatial attention task and 0.93 ± 0.18 s for the nonspatial attention task, with no significant difference between tasks (*P* = 0.27, Wilcoxon rank‐sum test). The rate of correct hits was 90.65 ± 4.17% for the spatial attention task and 93.77 ± 2.21% for the nonspatial attention task (*P* = 0.17, Wilcoxon rank‐sum test).

### Temporal Topographic Maps and ERSP Maps

**Figure 4:**
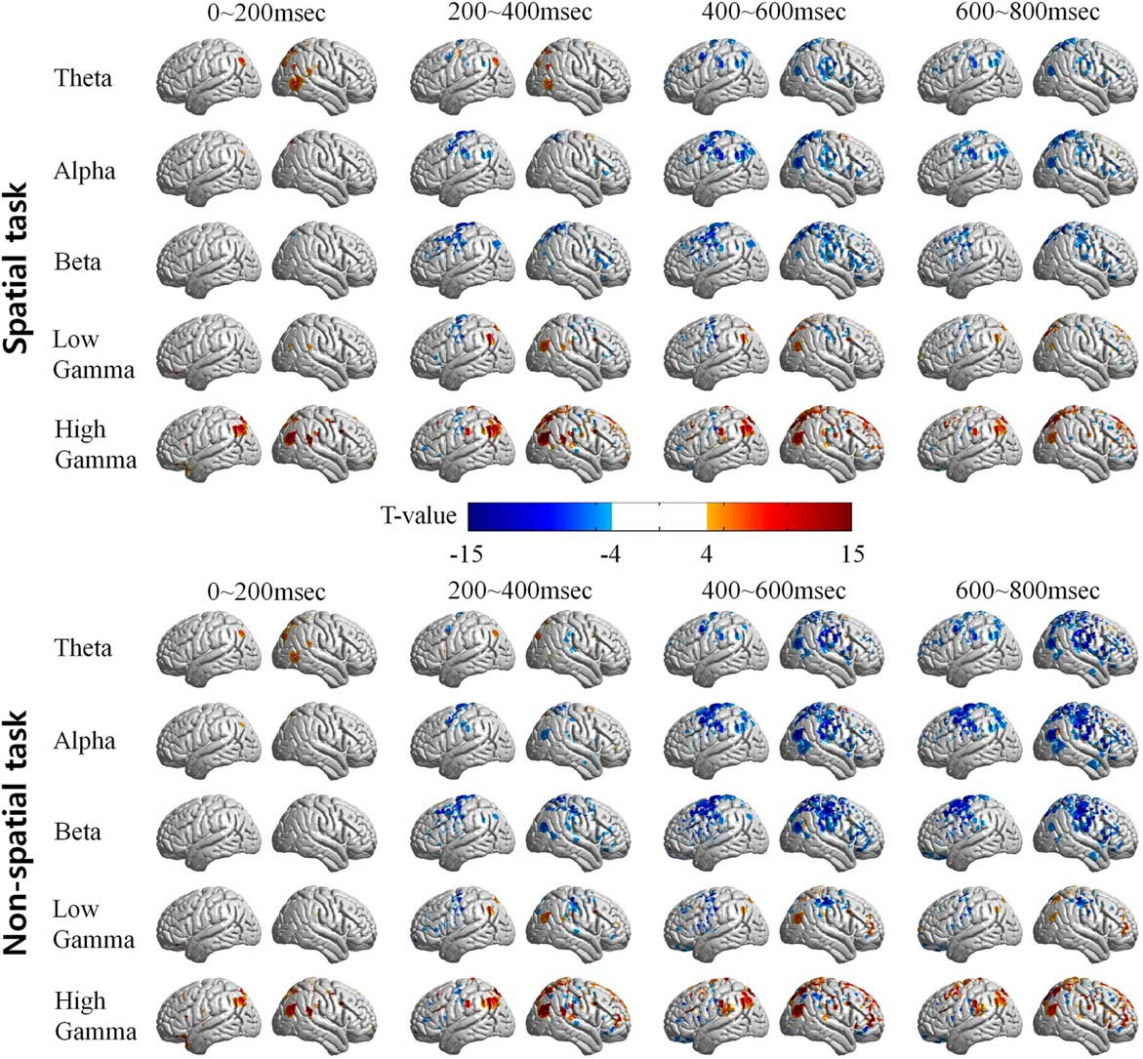
Spatiotemporal topography during the attention task determined by stimulus onset‐locked analysis. Significant differences in power between the task and the baseline are displayed on cortices following the stimulus during both spatial and nonspatial attention tasks. The activity altered over time but the most significant change occurred 400–600 ms after stimulus onset. [Color figure can be viewed in the online issue, which is available at http://wileyonlinelibrary.com.]

The spatiotemporal map after stimulus onset is shown in Figure 4 for both the spatial and the nonspatial attention task. The cortical distribution map of spatiotemporal dynamics from 0 to 800 ms showed significant ERD or ERS at 400–600 ms and 600–800 ms in both spatial and nonspatial attention tasks (Fig. 4). Generally, in both tasks, ERD clearly appeared in the low‐frequency bands, such as theta, alpha, and beta bands, whereas ERS was prominent in the high gamma band. Given the mean response times in the spatial and nonspatial attention tasks, the interval of 400–600 ms was selected for further analysis as it was considered to reflect attention engagement during the tasks [Wyart and Tallon‐Baudry, 2008].

#### Spatial attention

At 400–600 ms after the onset of the visual stimulus, low‐frequency bands showed maximal ERD in the right parietal lobe, followed by the left parietal lobe, bilateral frontal lobe, and right temporal lobe (Fig. 4). High‐frequency bands (high gamma) showed maximal ERS in the right parietal lobe, followed by the left parietal and right superior frontal lobes.

#### Nonspatial attention

At 400–600 ms after the onset of the visual stimulus, low‐frequency bands showed widespread ERD in the parietal lobe, more prominent on the right side than the left side, followed by the right frontal lobe, and right temporal lobe (Fig. 4). High‐frequency bands (high gamma) showed maximal ERS in the right parietal lobe, followed by the left parietal and right superior frontal lobes.

#### Brain areas with ERD or ERS in both spatial and nonspatial attention tasks

At 400–600 ms after the onset of the visual stimulus, the brain areas that showed ERD in both the spatial and nonspatial attention tasks were the bilateral parietal lobes (right > left), bilateral frontal lobe, and right temporal lobe in the low‐frequency bands. The brain areas that showed ERS in both tasks were the bilateral parietal lobes (right > left), right superior frontal lobe, and right temporal lobe (Fig. 4).

#### Brain areas with different ERD or ERS in spatial and nonspatial attention tasks

**Figure 5:**
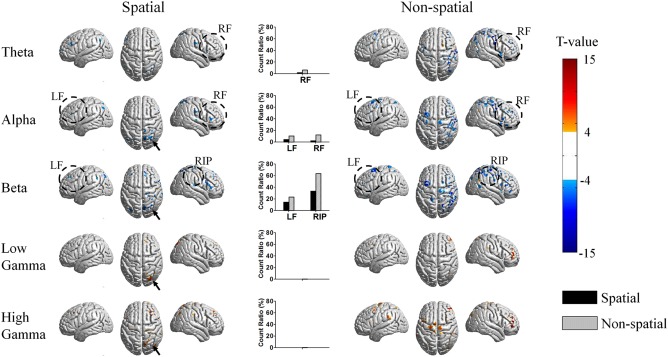
Cortical maps of selected electrodes determined by single electrode analysis 400–600 ms after stimulus onset. Brain areas showing ERD or ERS in only the spatial task or only the nonspatial attention task, respectively. The circle and bar graphs indicate areas that were statistically different between spatial and nonspatial attention tasks. The arrows point to parietal regions that had comparable activity in spatial and nonspatial attention tasks with statistically nonsignificant differences between the two tasks. [Color figure can be viewed in the online issue, which is available at http://wileyonlinelibrary.com.]

To identify differences in the cortical substrate of spatial and nonspatial attention tasks, those brain regions in which the signal power significantly changed in only the spatial attention task or only in the nonspatial attention task were selected, and are shown in Figure 5 along with the number of electrodes showing significant ERD and ERS.

The topographical map indicates that ERD in the right IPL (beta band), left frontal areas (alpha and beta bands), and the right frontal area (theta and alpha bands) was present in the nonspatial attention task but not the spatial attention task. In these regions, the number of electrodes showing significant ERD was greater in the nonspatial attention task than in the spatial attention task.

ERS was more widely observed in the nonspatial attention task than the spatial attention task. The topographic map indicates that ERS in the right SPL was greater in the spatial attention task than the nonspatial attention task. However, there was no significant difference in the number of electrodes showing ERS in the spatial and nonspatial attention tasks.

### Electrode Counting

**Figure 6:**
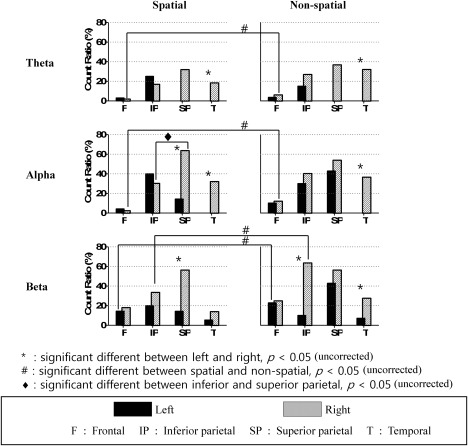
Percentage of electrodes showing ERD at 400–600 ms after stimulus onset in each region of interest determined by stimulus onset‐locked analysis. The count ratio over the low‐frequency (theta, alpha, and beta) bands shows right dominance for both spatial and nonspatial attention tasks. On the right side, the superior parietal region was the most active region in the spatial attention task.

The fraction of electrodes showing significant ERD or ERS 400–600 ms after the onset of the visual stimulus compared with the baseline state in each frequency band during the spatial and nonspatial attention tasks are shown in Figures 5 and 6.

#### Comparison between spatial and nonspatial attention tasks

**Figure 7:**
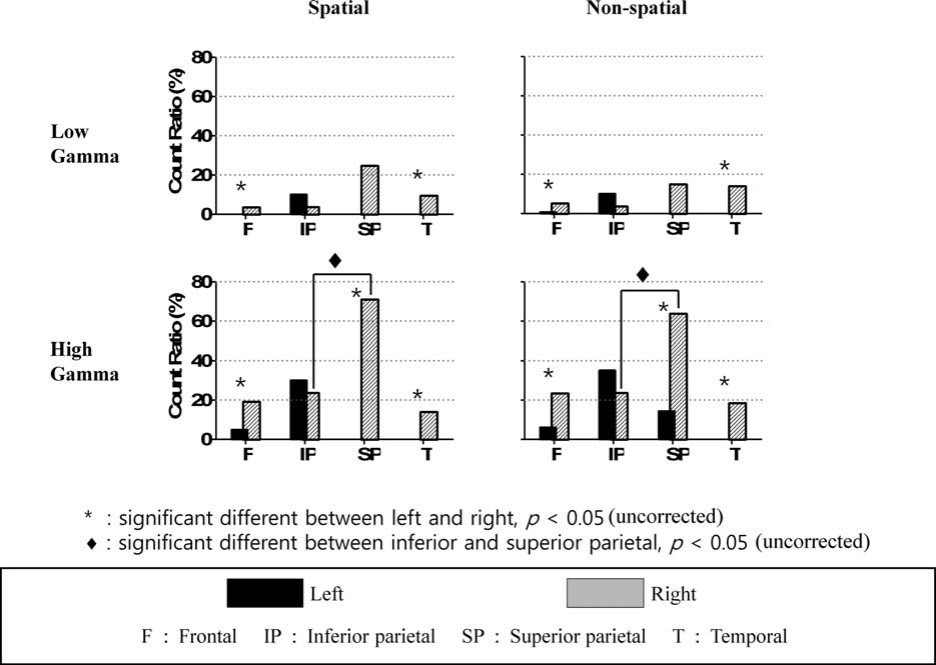
Percentage of electrodes showing ERS at 400–600 ms after stimulus onset in each region of interest determined by stimulus onset‐locked analysis. In the high‐frequency (low and high gamma) bands, the count ratio shows dominance in the superior parietal region for both spatial and nonspatial attention tasks.

ERD was more widely observed in the nonspatial attention task than in the spatial attention task. The fraction of electrodes showing significant ERD was greater in the nonspatial attention task than the spatial attention task for the right frontal lobe (theta and alpha bands), left frontal lobe (alpha, beta, low gamma, and high gamma bands), and the right IPL (beta and high gamma bands) (Figs. 5 and 6). The fraction of electrodes showing significant ERS was not significantly different between the two tasks for any brain region (Fig. 7).

#### Comparison of left–right asymmetry in ERD and ERS

In the spatial attention task, there was significant left–right asymmetry in the fraction of electrodes showing ERD, with more electrodes in the right temporal lobe (theta and alpha bands) and right SPL (alpha and beta bands) showing ERD compared with the corresponding regions in the left hemisphere. In the nonspatial attention task, there was also significant left–right asymmetry in the fraction of electrodes showing ERD, with more electrodes in the right temporal lobe (theta, alpha, and beta bands) and right IPL (beta band) showing ERD compared with the corresponding regions in the left hemisphere (Fig. 6).

For both tasks, there was significant left–right asymmetry in the fraction of electrodes showing ERS, with more electrodes in the right frontal and temporal lobes (low gamma and high gamma bands) and right SPL (high gamma band) showing ERS compared with the corresponding regions in the left hemisphere (Fig. 7).

#### Comparison between right IPL and SPL

In the spatial attention task, the fraction of electrodes showing ERD in the alpha frequency band was greater in the right SPL than in the right IPL. By contrast, in the nonspatial attention task, there was no significant difference in the fraction of electrodes showing ERD between the right IPL and right SPL for any frequency band (Fig. 6). In both tasks, the fraction of electrodes showing ERS in the high gamma band was significantly higher in the right SPL than in the right IPL (Fig. 7).

### Comparison between Target and Nontarget Responses

**Figure 8:**
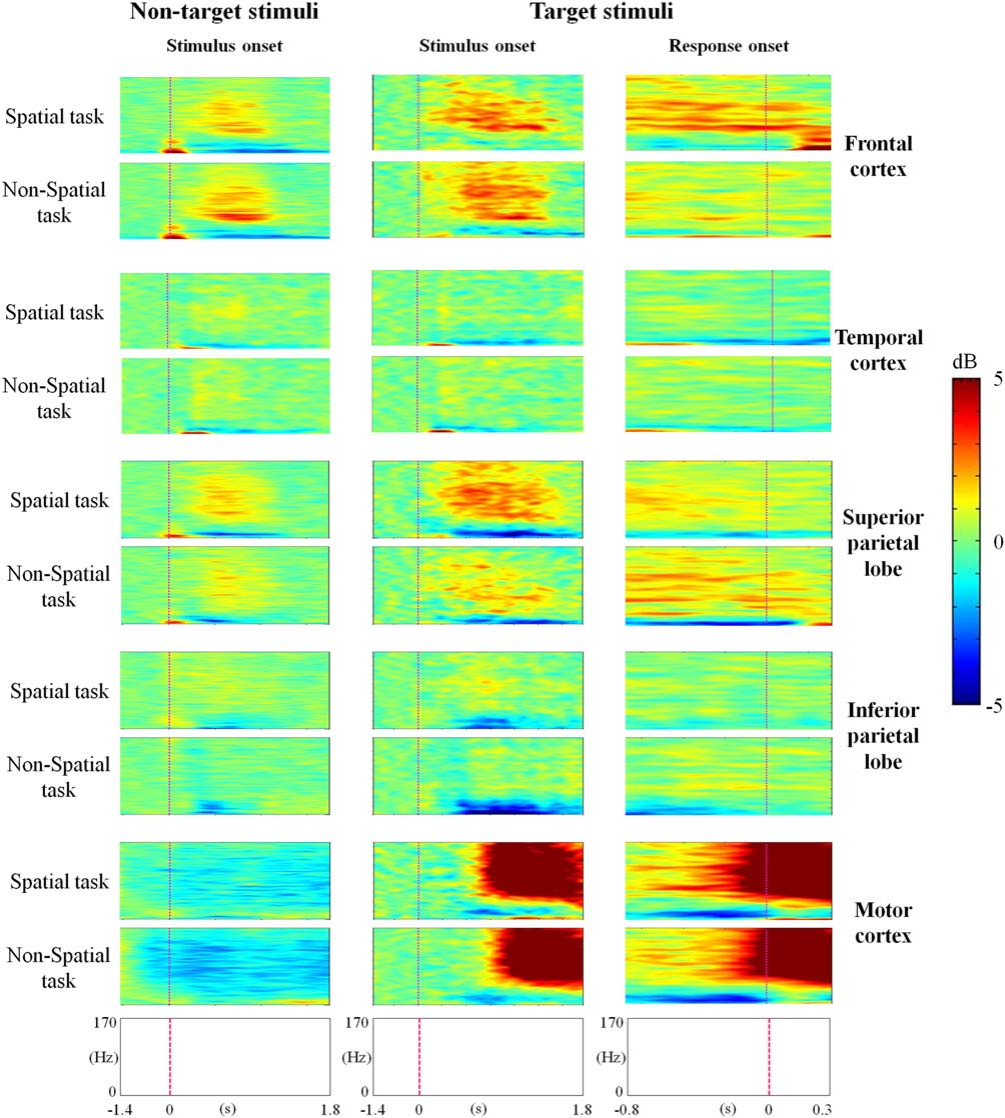
ERSP maps for specific electrodes. ERSP maps show that the activation was not a motor response. [Color figure can be viewed in the online issue, which is available at http://wileyonlinelibrary.com.]

We compared the trials with a motor response (target stimuli) to trials without a motor response (nontarget stimuli) to ensure that the iEEG signals attributed to spatial or nonspatial attention were not linked to the motor response. Electrodes over the motor cortex showed ERD in the lower frequency bands and ERS in the higher frequency bands that were linked to the motor response after presentation of target stimuli, but they did not show ERD or ERS for nontarget stimuli. ERD and ERS in the right SPL and IPL, frontal (nonmotor area), and temporal lobes were observed before motor responses and were not time‐locked to the motor response, suggesting that ERD or ERS in the parietal, frontal (nonmotor area), and temporal lobes were not linked to the motor response (Fig. 8).

## DISCUSSION

In this study, we used iEEG signals to investigate the neural correlates of spatial and nonspatial attention over time. Stimuli for the spatial and nonspatial attention tasks were identical and behavioral performance, quantified by reaction time and error rate, did not significantly differ across the tasks.

### Similarities and Differences between the Two Tasks

Both common and different brain areas were engaged during the spatial and nonspatial attention tasks. In this study, the only difference between spatial and nonspatial tasks was what to attend to. The visual stimuli and motor response of the participant were identical for both conditions. Based on this design, a large portion of the cognitive processes were identical, leading to the overlapping activity between the tasks. In both tasks, activity was observed bilaterally in the parietal cortex, although more prominently in the right parietal lobe than the left parietal lobe, and in frontal and temporal lobe regions. These findings support previous results showing involvement of frontoparietal regions, including the posterior parietal cortex and the superior frontal lobe, in both spatial and nonspatial attention [Corbetta and Shulman, 2002; Giesbrecht et al., 2003].

An important aim of this study was to determine whether there are differences in the brain regions associated with spatial and nonspatial attention. When participants performed the spatial attention task, significant ERD and ERS were observed, mainly in the right parietal lobe, especially the right SPL. By contrast, when participants performed the nonspatial attention task, significant ERD and ERS changes were more widely distributed, and were observed bilaterally in the parietal lobes (maximal on the right side), the bilateral frontal region, and the right temporal regions. Additionally, ERD was prominent in the right SPL during the spatial attention task, but in the right IPL during the nonspatial attention task. The ERD and ERS are unlikely to be due to a motor response because they were observed before motor responses and were not time‐locked to motor response. Overall, these findings suggest that the right SPL and IPL might play different roles in spatial and nonspatial attention tasks. To the best of our knowledge, this is the first study to use iEEG to show how spatial and nonspatial attention tasks, performed using the same stimuli, engage common as well as different brain networks.

In this study, ERD showed significant hemispheric asymmetry in the spatial attention task, whereby the fraction of electrodes showing ERD was greater in the right hemisphere than in the left hemisphere. By contrast, there was no significant hemispheric asymmetry in the nonspatial attention task. These findings are consistent with the view that spatial attention is especially associated with the involvement of brain networks in the right hemisphere, including the right parietal lobe, whereas nonspatial attention relies on wider bilateral hemispheric brain networks. This might explain why hemispatial neglect patients with right parietal lesions exhibited a vigilance decrement only during a spatial attention task [Malhotra et al., 2009].

### Role of the Posterior Parietal Cortex in Spatial and Nonspatial Attention

The posterior parietal cortex has traditionally been considered to have a special role in spatial functions. The posterior parietal cortex is viewed as the target of the dorsal visual stream of cortical pathways that originate in the primary visual cortex and project to the parietal lobe [Milner and Goodale, 1995; Nachev and Husain, 2006; Ungerleider and Mishkin, 1982]. Electrophysiological recordings from awake behaving monkeys [Andersen and Buneo, 2002] leave little doubt that representation of space is an important function of this region. However, there is also evidence in humans for a role of the posterior parietal cortex in selective attention tasks that do not require spatial shifts of attention [Husain and Rorden, 2003; Vandenberghe et al., 2001a]. Our results show maximal ERD and ERS in the parietal, especially the right parietal, cortex during both spatial and nonspatial attention tasks, and are consistent with previous studies that have suggested that the right parietal lobe plays a crucial role in both spatial and nonspatial attention [Husain and Rorden, 2003; Vandenberghe et al., 2001a].

In this study, the fraction of electrodes in the right SPL that showed ERD in the alpha band during the spatial attention task was greater than that in the right IPL, but there was no such difference between the right SPL and IPL in the nonspatial attention task. A comparison of the cortical map between spatial and nonspatial attention tasks reveals that regions that showed significant ERD only in the spatial attention task were predominantly located in the right SPL, and regions that showed significant ERD only in the nonspatial attention task were located in the right IPL. These findings suggest different roles for the right SPL and IPL in spatial and nonspatial attention tasks. Previous reports, including several functional imaging studies, have revealed greater involvement of the SPL and intraparietal sulcus in spatial shifts of attention and involvement of the right IPL in tasks that are not necessarily spatially lateralized as well as in detecting salient novel events [Adler et al., 2001; Corbetta and Shulman, 2002; Husain and Nachev, 2007; Husain and Rorden, 2003; Johannsen et al., 1997; Nachev and Husain, 2006; Vandenberghe et al., 2012]. Lesions of the SPL do not lead to impairments in nonspatial selective attention tasks [Shapiro et al., 2002; Vandenberghe et al., 2001b]. By contrast, lesions of the IPL in patients who do not have neglect lead to impairments in tests that involve the counting of nonlateralized monotonous auditory tones and the detection of a visual target at the center of a screen or of tactile stimuli presented to either hand [Rueckert and Grafman, 1996, 1998]. This study provides direct electrophysiological evidence for different roles of the right SPL and IPL during spatial and nonspatial attention tasks.

### The Roles of Low‐ and High‐Frequency Oscillations

In this study, we showed ERD in low‐frequency bands and ERS in high‐frequency bands during both spatial and nonspatial attention tasks. Low‐frequency oscillations, such as those in the alpha and theta frequency bands, are considered to be an expression of a lack of attention, and suppression of such activities is an active mechanism during the deployment of attention [Shulman et al., 2002; Thut et al., 2006], such as release from inhibition [Klimesch, 1999]. We found that both spatial and nonspatial attention modulated oscillations at low frequencies, such as in the theta and alpha bands, in both hemispheres, including the right parietal regions, suggesting that decreased oscillatory power in low‐frequency bands serves attentional engagement in the right parietal cortex.

Several studies have suggested that gamma band synchronization has a pivotal function in attention. Gamma band signals are considered to be important for the transient integration of neural activities [Luo et al., 2009]. The power of gamma activity is increased during cognitive processes [Corbetta and Shulman, 2002]. Previous studies have reported associations between attention and gamma frequency synchronization to integrate neural assemblies associated with a specific sensory object [Jensen et al., 2007], and gamma band changes have been reported across the visual cortex, frontal eye fields, SPL, and dorsal lateral prefrontal cortex [Buschman and Miller, 2007; Ossandon et al., 2012]. The decreased power in low‐frequency bands and increased power in high‐frequency bands in this study is compatible with these findings and shows that common brain areas were actively involved during the two tasks.

### Adequateness of the Experimental Paradigm

In the present experiments, all participants used their right hand to press a keyboard button when they saw the target stimulus. ERD and ERS observed in the left somatosensory and motor cortices can be attributed to the motor response as they were time‐locked to the response and observed before and during execution of unilateral movements after 600 ms. This finding is consistent with the fact that the lateralization of spectral power decreases in the sensorimotor regions contralateral to the responding hand [Pfurtscheller and Lopes da Silva, 1999].

It might be argued that more widespread ERD and ERS responses in the nonspatial attention task can be attributed to working memory demand. However, memory demands for the spatial and nonspatial attention tasks in this study were low, because participants only needed to keep two spatial targets online to perform the task accurately [Malhotra et al., 2009]. Target identities remained static throughout the duration of the task, minimizing requirements for the manipulation of information, and similar paradigms have been used in previous spatial and nonspatial attention tasks [Malhotra et al., 2009]. Moreover, there were no significant differences in the average reaction time and correct hit rate between the two tasks, suggesting that there were no differences in task difficulty.

### Limitations

This study has several limitations. First, electrodes showing infrequent or rare interictal epileptiform discharges were included and only electrodes showing frequent interictal epileptiform discharges or located on structural brain lesions were excluded. We do not think that the infrequent epileptiform discharges distorted our results because they appeared randomly across the two tasks. Second, participants were presurgical epilepsy patients. Thus, the placement of the electrodes was determined by clinical need and subdural electrodes did not cover the whole brain area. As a consequence, the results of this study do not fully encompass the attentional networks. In particular, the low number of electrodes in the left SPL needs to be considered when interpreting the results. We do not consider the use of antiepileptic drugs to be a potential confounding factor in this study because we compared iEEG data between spatial and nonspatial attention tasks performed by the same participants.

## CONCLUSION

Our current findings suggest that nonspatial attention is associated with engagement of more widespread brain networks than spatial attention, and that spatial attention is more prominently associated with right hemispheric activity, particularly in the parietal lobe. Within the parietal lobe, the spatial attention task evoked ERD or ERS in the right SPL, whereas the nonspatial attention task evoked ERD in the right IPL.

## Supporting information

Supporting Information Figure 1.

Supporting Information Figure 2.

Supporting Information Figure 3.

Supporting Information Figure 4.
