# Supplementary figures and images for: Neural correlates of spatial and nonspatial attention determined using intracranial electroencephalographic signals in humans
Source: Hum Brain Mapp. 2016 Apr 29;37(8):3041–54. doi: 10.1002/hbm.23225 (PMC5025724; doi:10.1002/hbm.23225)

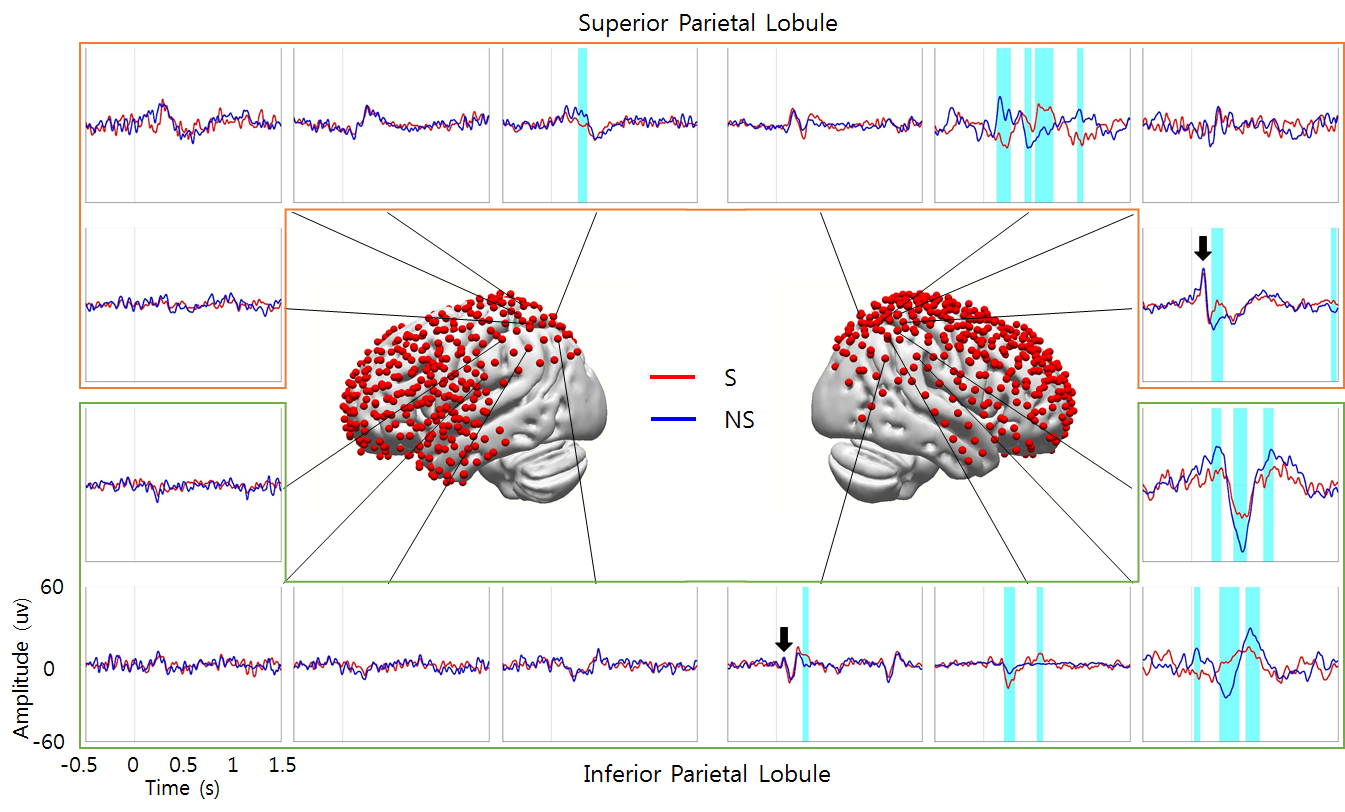

Supplement: Supplementary file 1 — Supporting Information Figure 1. [file HBM-37-3041-s001.tif]

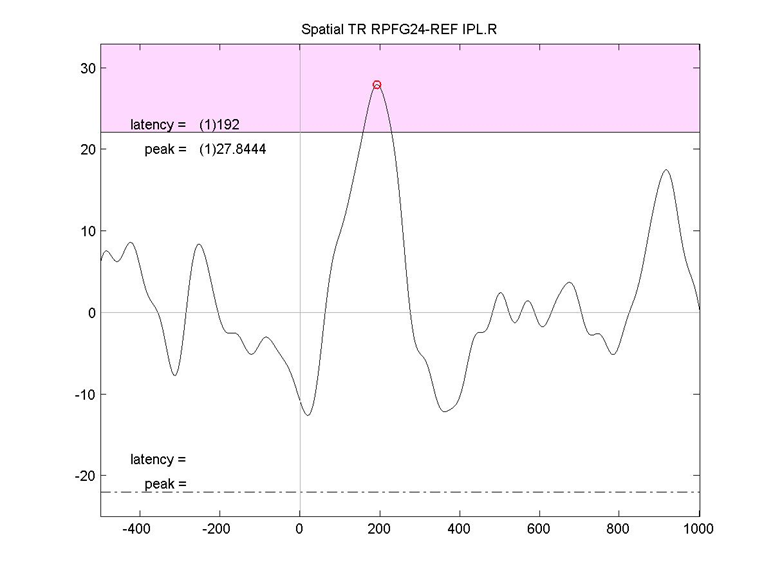

Supplement: Supplementary file 2 — Supporting Information Figure 2. [file HBM-37-3041-s002.tif]

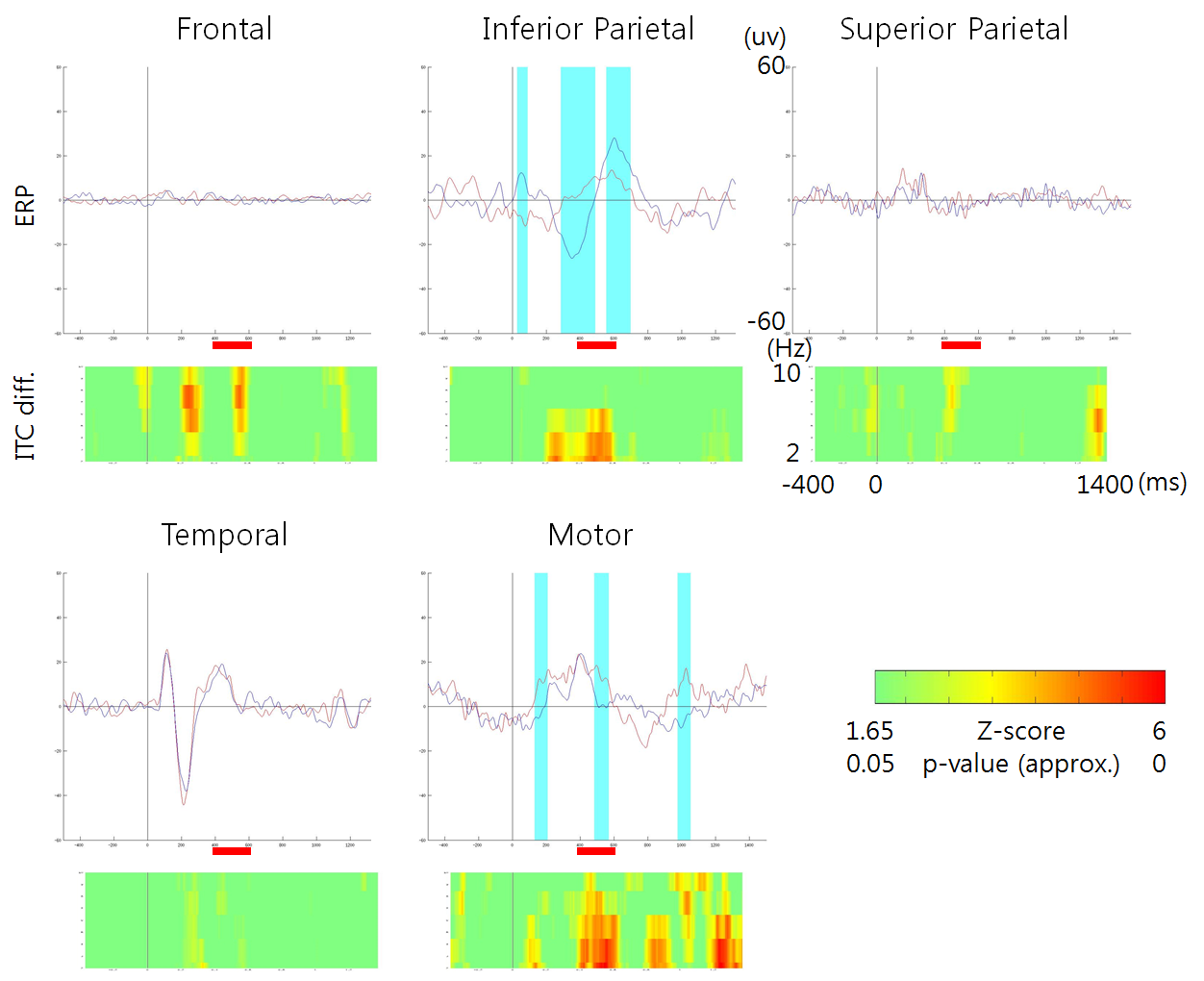

Supplement: Supplementary file 3 — Supporting Information Figure 3. [file HBM-37-3041-s003.tif]

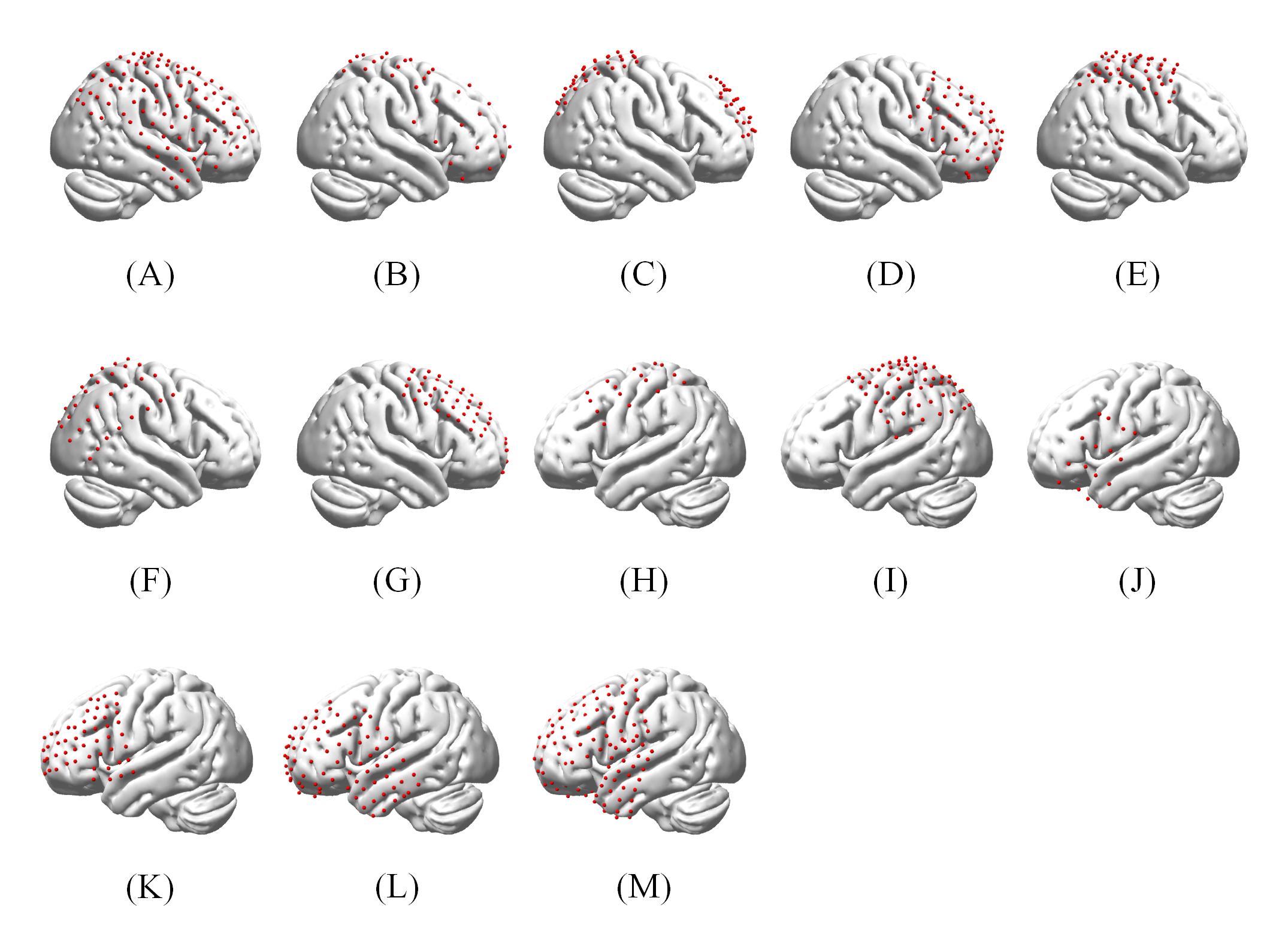

Supplement: Supplementary file 4 — Supporting Information Figure 4. [file HBM-37-3041-s004.tif]
